# Supplementary material for: Rhenium(I) Block Copolymers Based on Polyvinylpyrrolidone: A Successful Strategy to Water-Solubility and Biocompatibility
Source: Molecules. 2023 Jan 1;28(1):348. doi: 10.3390/molecules28010348 (PMC9822124; doi:10.3390/molecules28010348)
Supplement: Supplementary file 1 [file molecules-28-00348-s001.zip › molecules-2124933-SM.pdf]

Electronic Supporting Information for the article

Rhenium(I) Block-Copolymers Based on Polyvinylpyrrolidone: Successful Strategy to Water-Solubility and Biocompatibility

Kristina S. Kisel [1], Vadim A. Baigildin [1], Anastasia I. Solomatina [1], Alexey I. Gostev [2], Eugene V. Sivtsov [2], Julia R. Shakirova [1,*] and Sergey P. Tunik [1,*]

[1] Institute of Chemistry, Saint-Petersburg State University, Universitetskii pr., 26, 198504 St. Petersburg, Russia

[2] Department of physical chemistry, Saint-Petersburg State Institute of Technology (Technical University), Moskovskiy pr. 26, 190013 St. Petersburg, Russia

* Correspondence: y.r.shakirova@spbu.ru (J.R.S.), sergey.tunik@spbu.ru (S.P.T.)

Content

Part 1. NMR spectroscopy, ESI mass-spectrometry, XRD-analysis of complexes Re1−Re4.. 2

Part 2. NMR spectroscopy and Dynamic light scattering analysis of p(VP-l), p(VP-h) and p(VP-l/h-Re1)−p(VP-l/h-Re4) 6

Part 3. Electronic absorption spectra of complexes Re1−Re4.................................... 10

Part 4. Photophysical properties of p(VP-l/h-Re1)−p(VP-l/h-Re4)............................. 11

Part 5. Cell experiments for Re2 13

Part 1

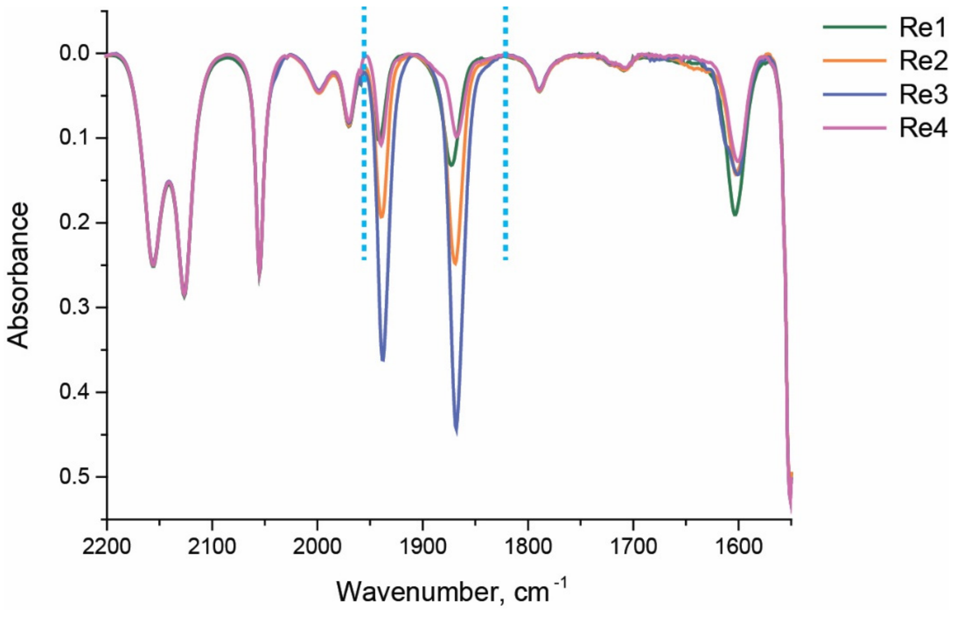

Figure S1. IR spectra of Re1−Re4 (CD2Cl2).

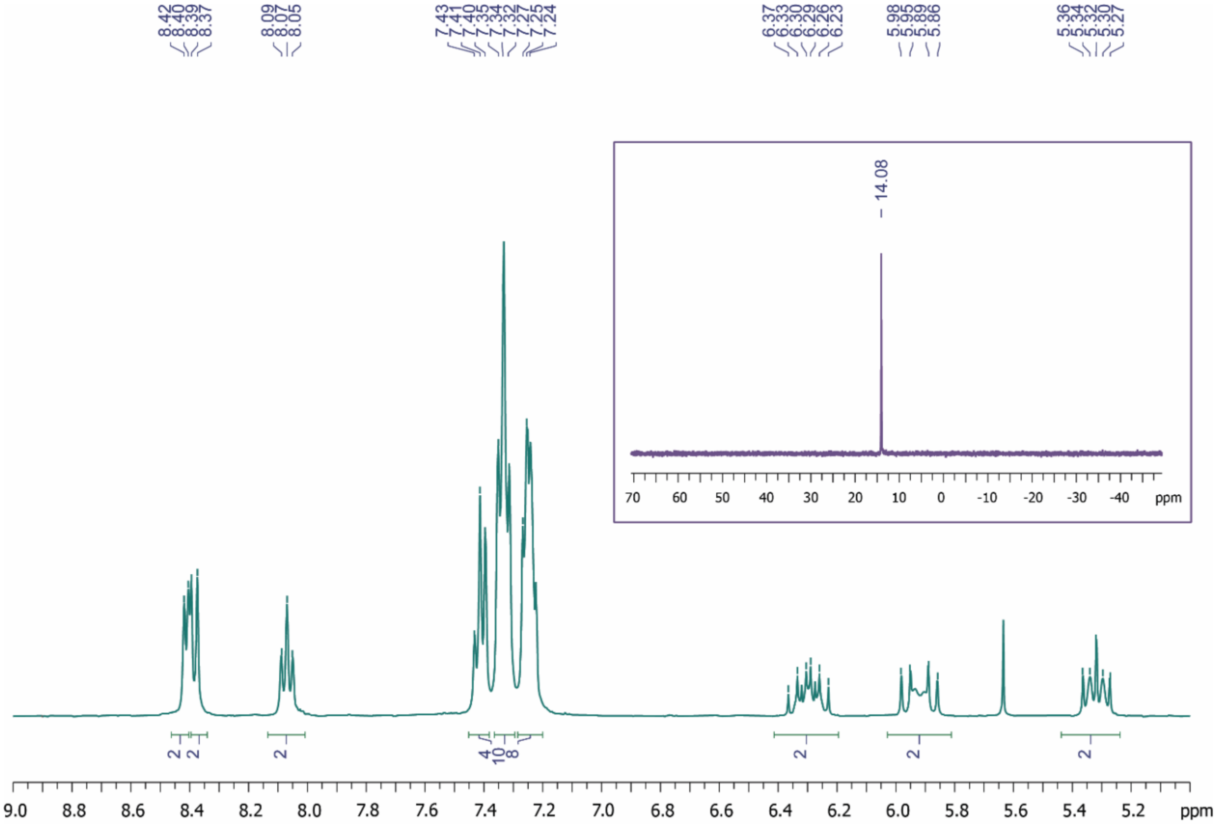

Figure S2. ^{1}H NMR and ^{31}P{H} spectra of **Re1** (acetone-d_6, 298K).

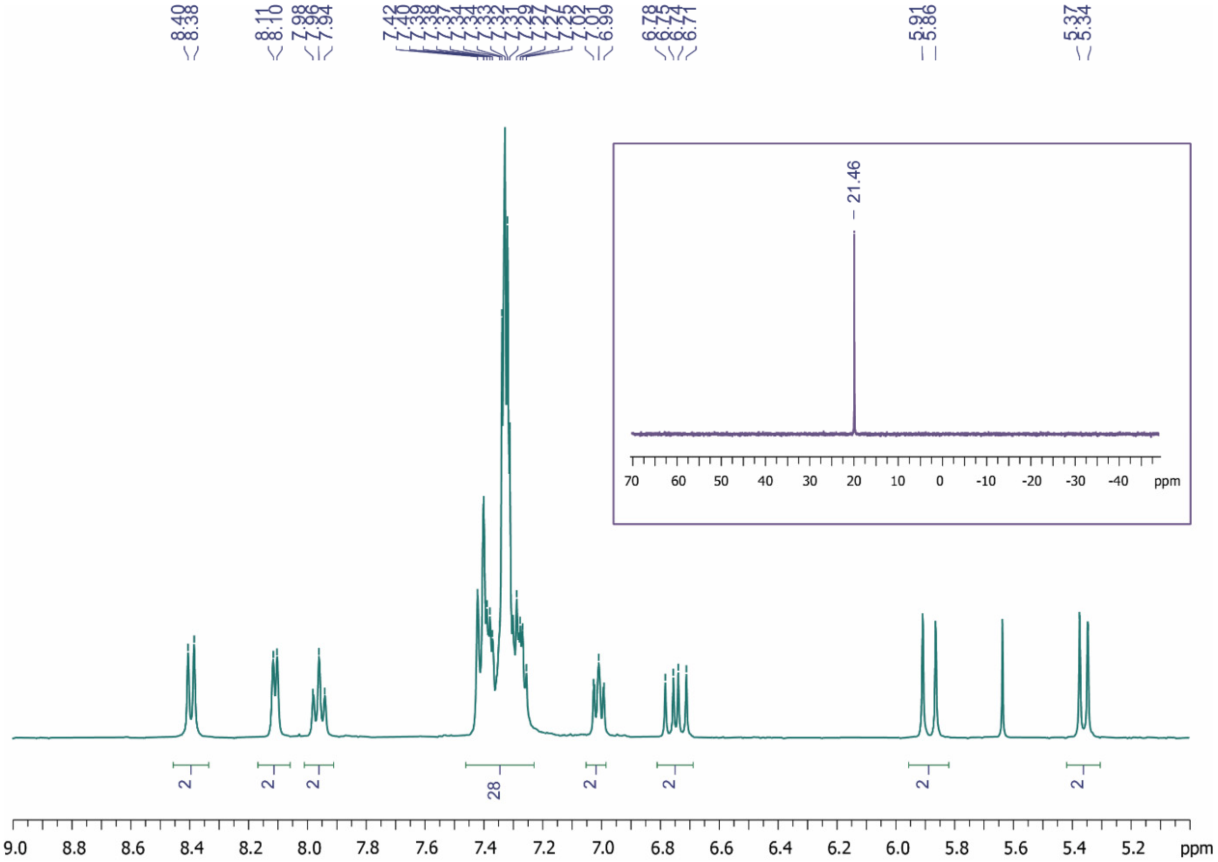

Figure S3. ^{1}H NMR and ^{31}P{H} spectra of **Re2** (acetone-d_6, 298K).

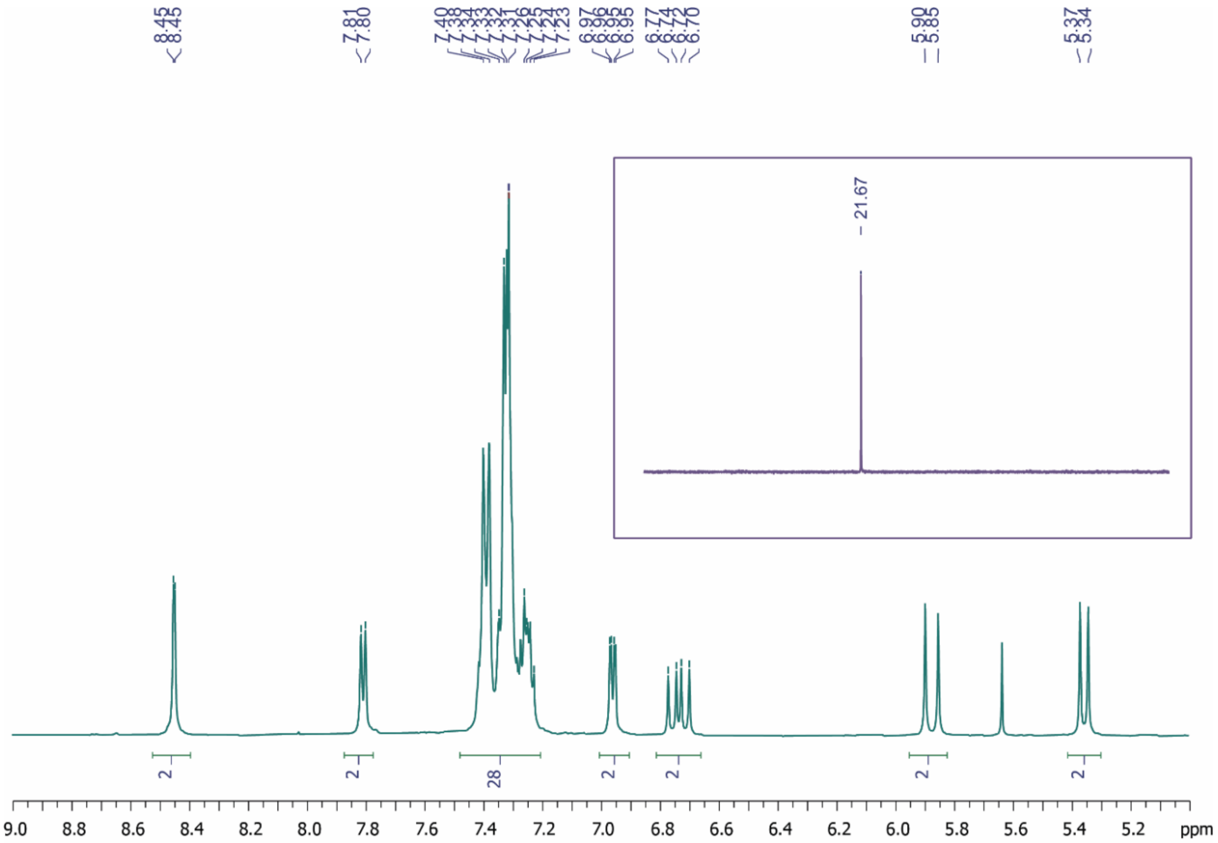

Figure S4.^{1}H NMR (aromatic region) and ^{31}P{H} spectra of **Re3** (acetone-d_6, 298K).

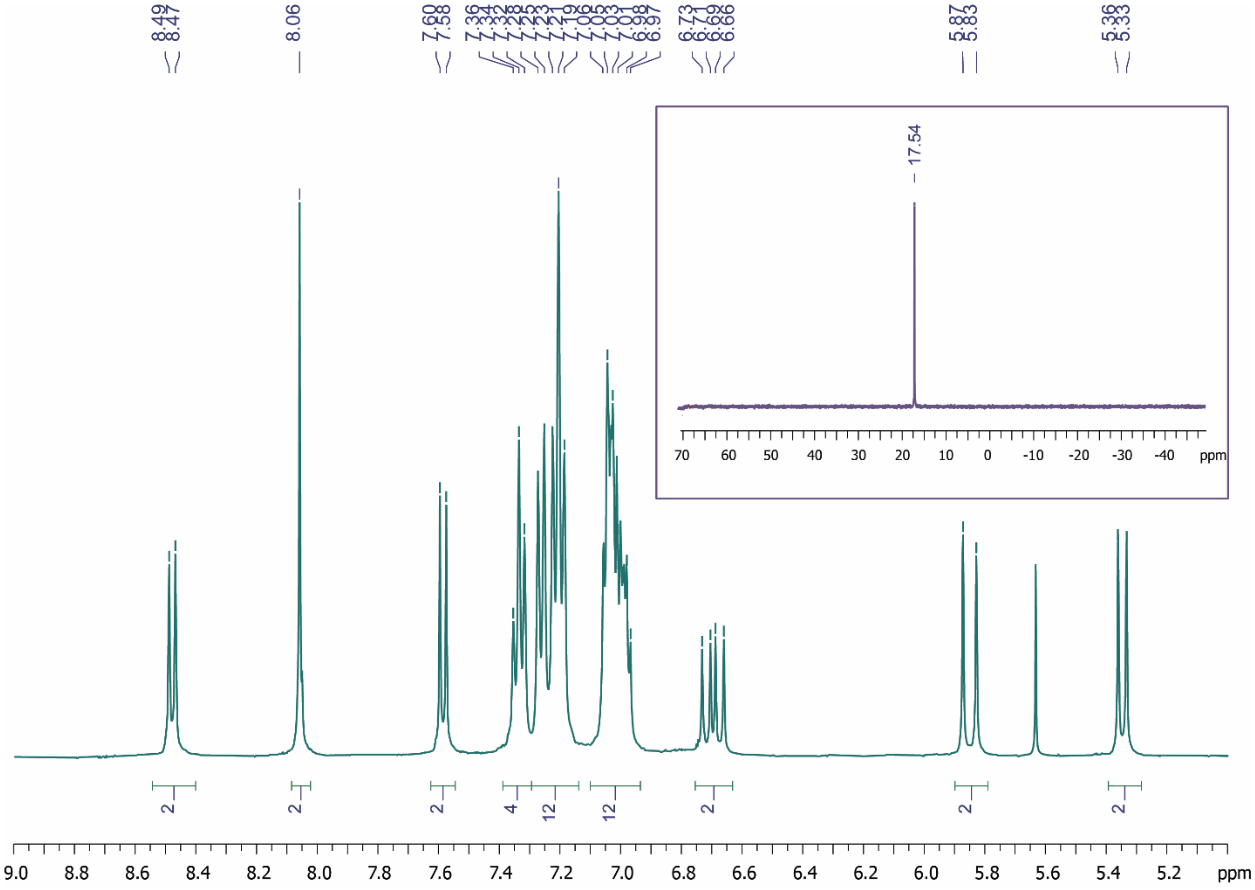

Figure S5. ^{1}H NMR (aromatic region) and ^{31}P{H} spectra of **Re4** (acetone-d_6, 298K).

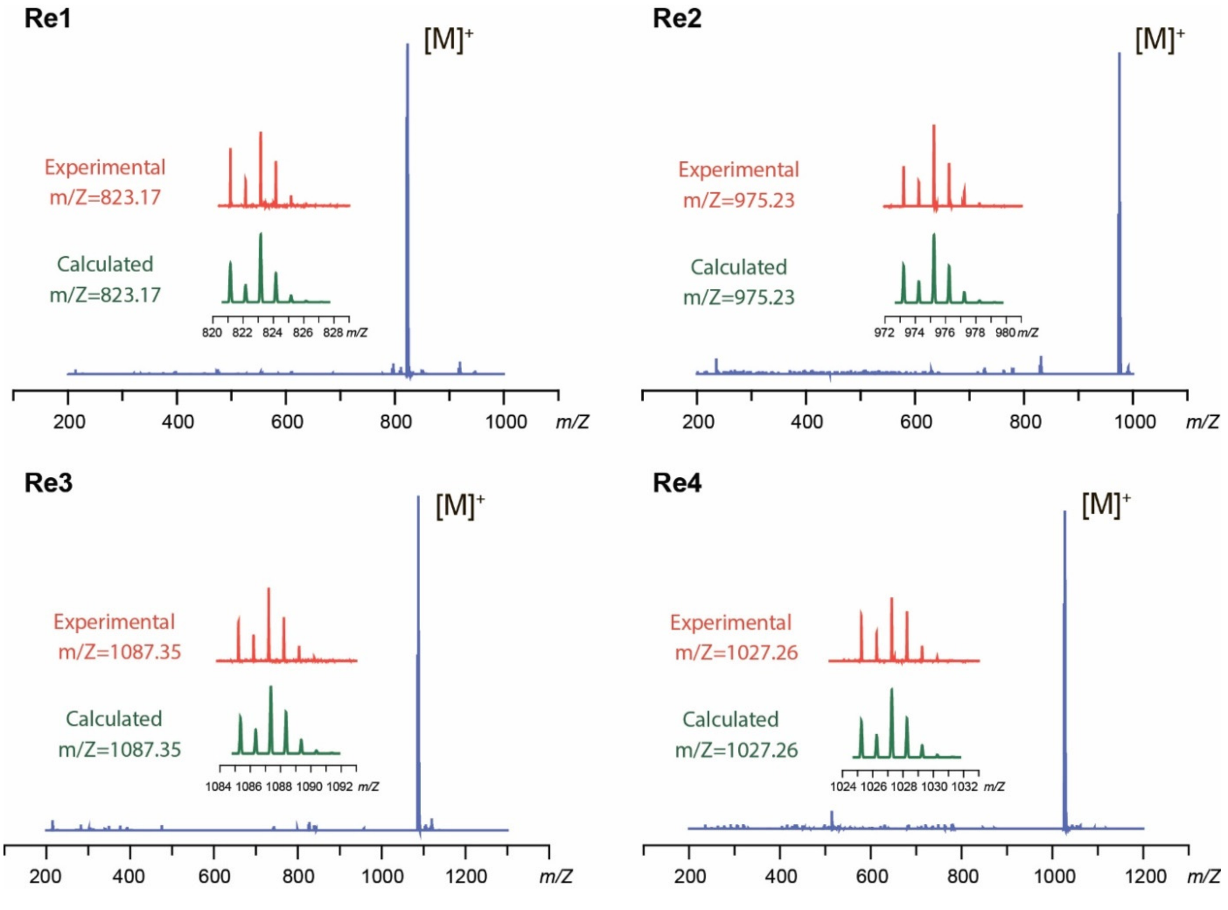

Figure S6. ESI^{+} mass spectra of **Re1–Re4**.

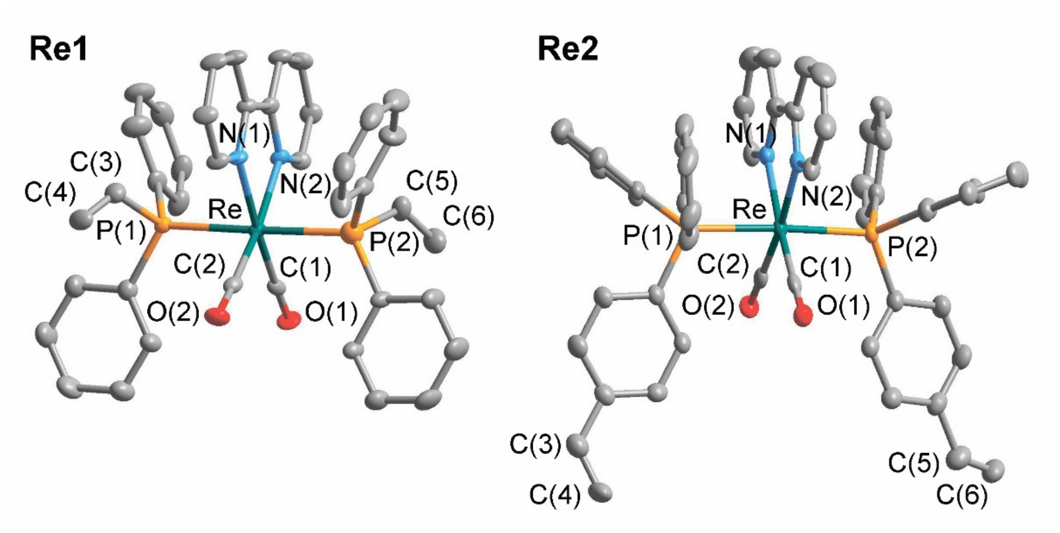

Table S1. Selected bond lengths and angles for **Re1** and **Re2**.

	**Re1**	**Re2**
Bond lengths, Å		
Re-N1	2.172(5)	2.175(4)
Re-N2	2.177(4)	2.171(5)
Re-P1	2.4079(15)	2.4347(12)
Re-P2	2.4047(15)	2.4218(12)
Re-C1	1.905(6)	1.910(6)
Re-C2	1.921(6)	1.904(6)
C3-C4	1.295(11)	1.298(10)
C5-C6	1.304(12)	1.292(11)
Bond angles, °		
N1-Re-N2	74.58(18)	74.96(17)
C(1)-Re-C(2)	91.5(2)	93.0(2)
P(1)-Re1-P(2)	178.35(5)	176.53(4)

Part 2

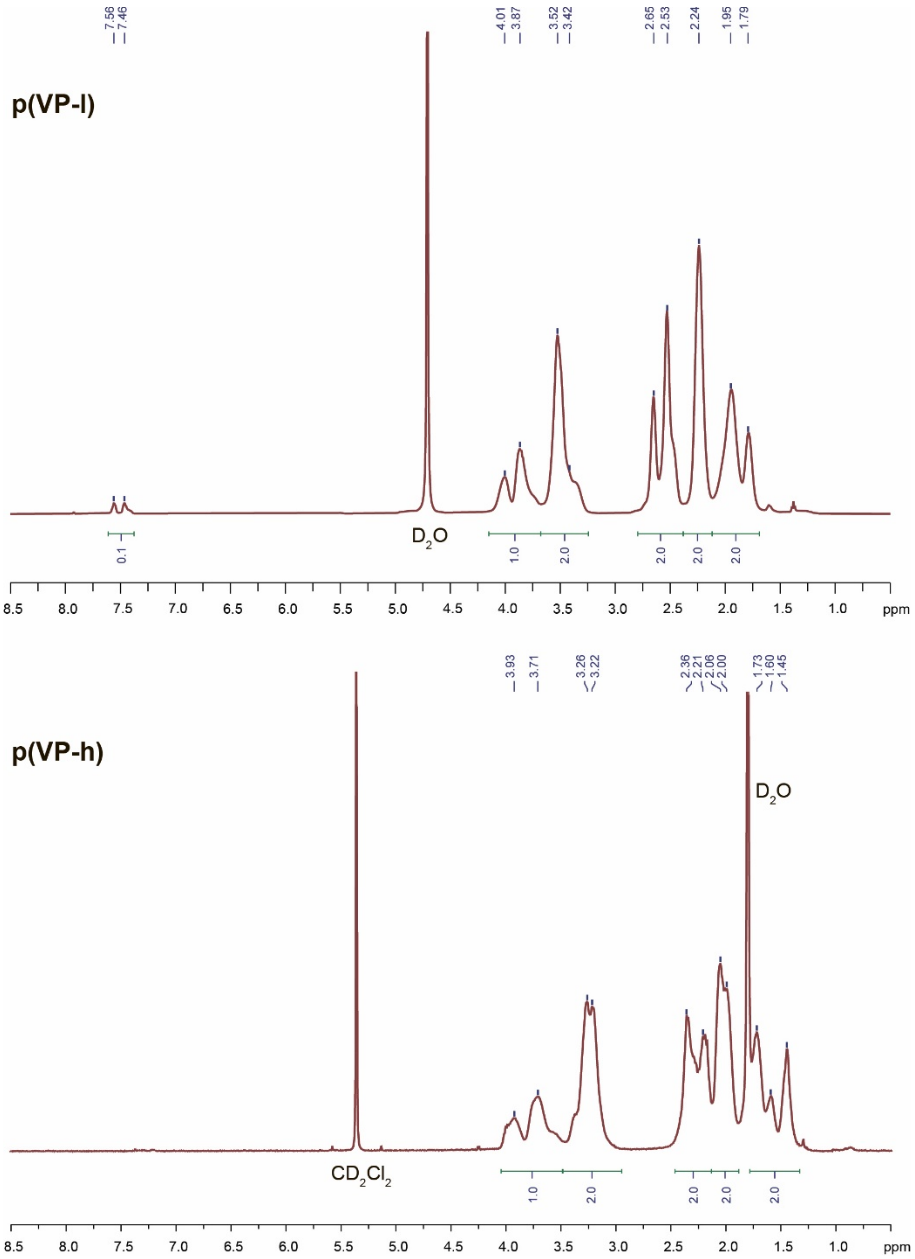

Figure S7. ^{1}H NMR spectra of **p(VP-l)** (D_2O, 298K) and **p(VP-h)** (CD_2Cl_2, 298K).

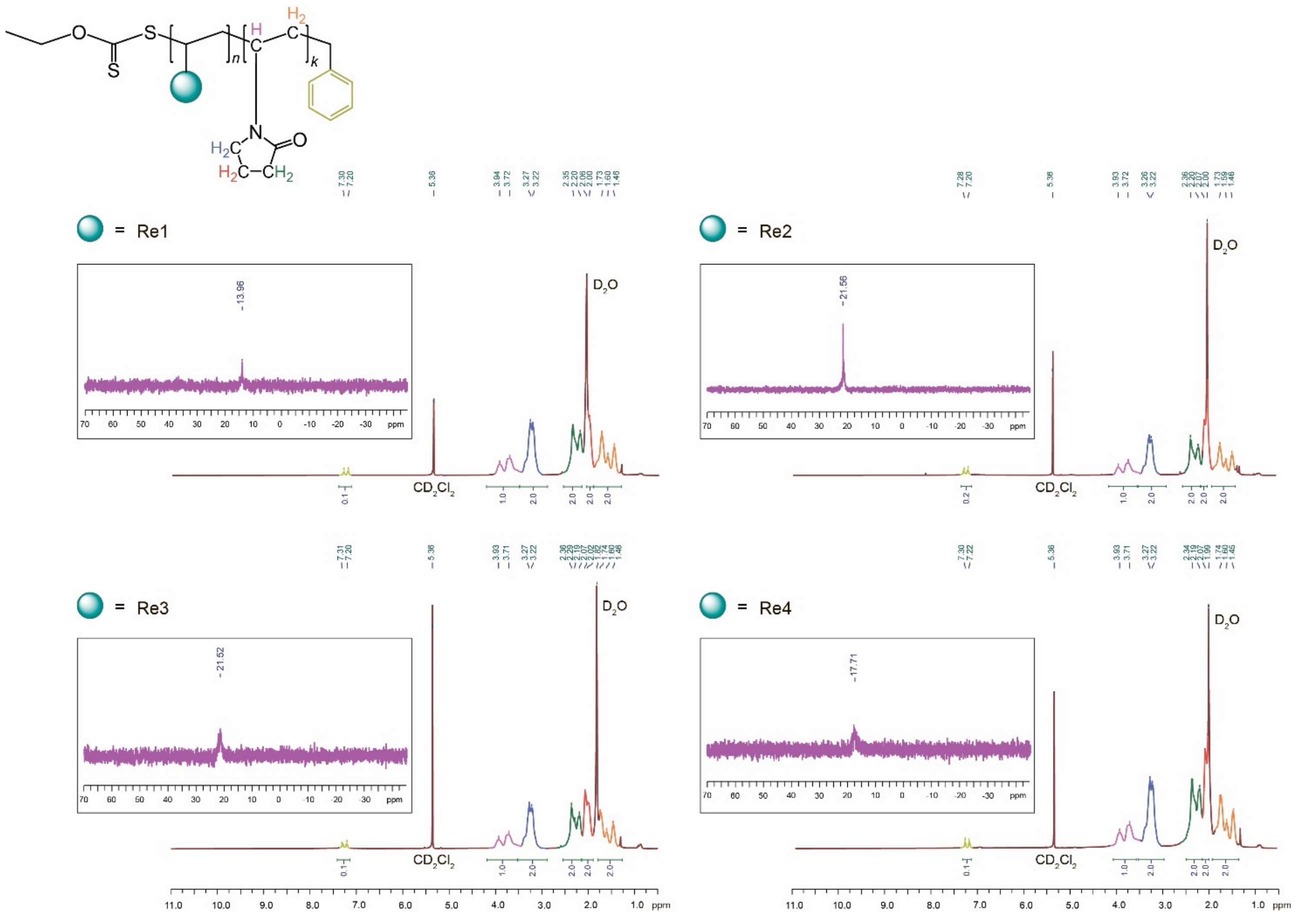

Figure S8. ^{1}H NMR and ^{31}P{H} spectra of **p(VP-l-Re)** (CD_2Cl_2, 298K).

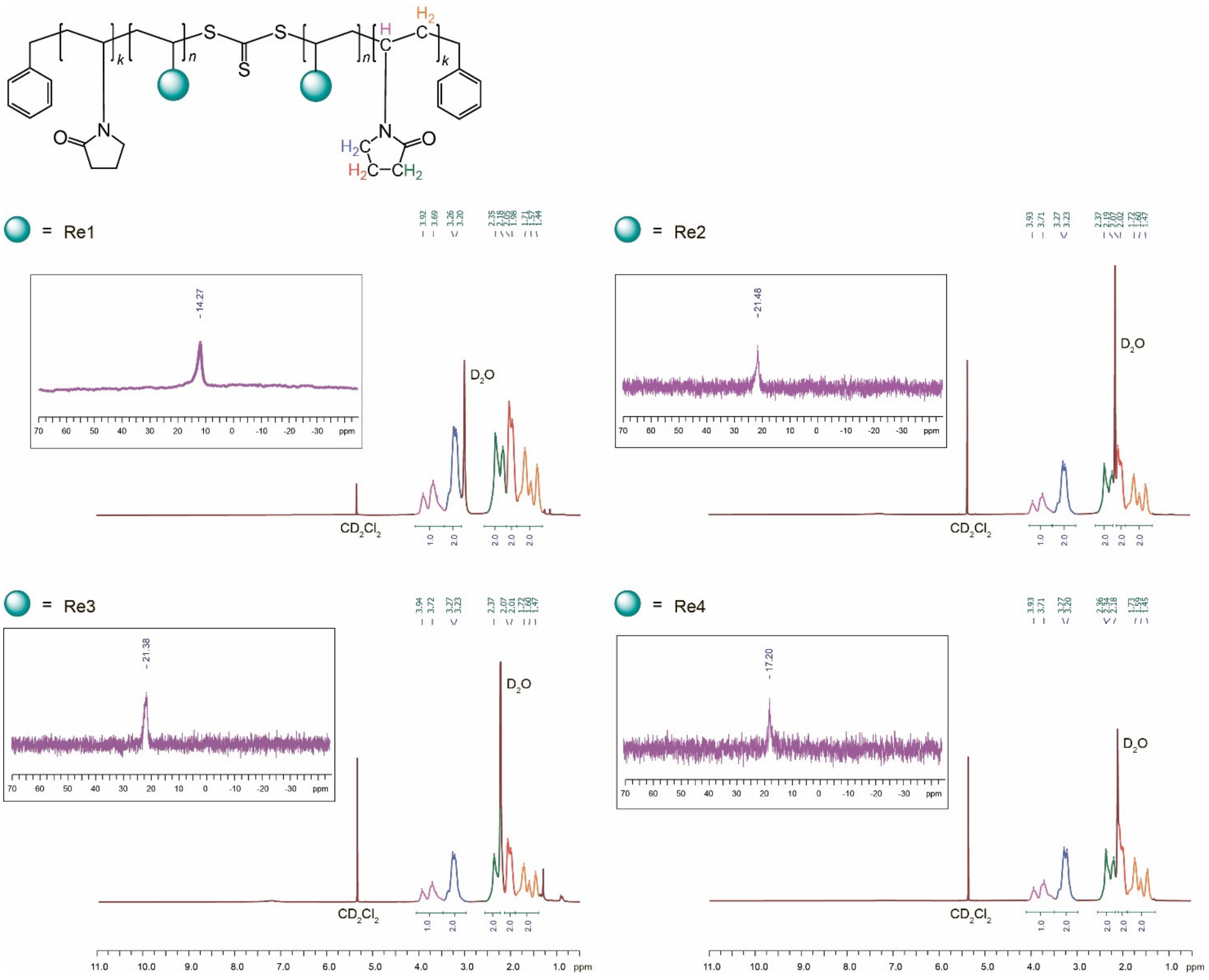

Figure S9. ^{1}H NMR and $^{31}P\{H\}$ spectra of **p(VP-h-Re)** (CD_2Cl_2, 298K

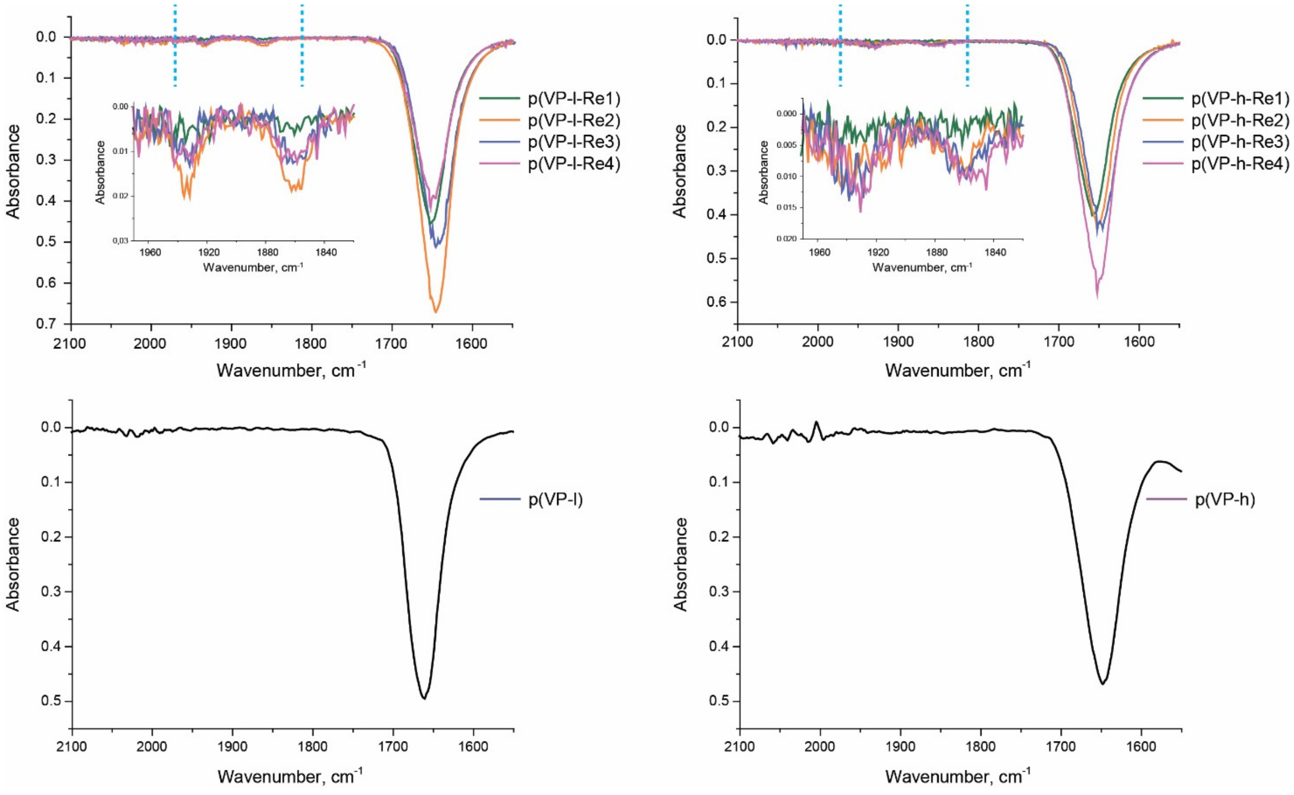

Figure S10. Top: IR spectra of the block-copolymers p(VP-l/h-#) (solid samples). Bottom: IR spectra of the polymers p(VP-l/h) for comparison (solid samples).

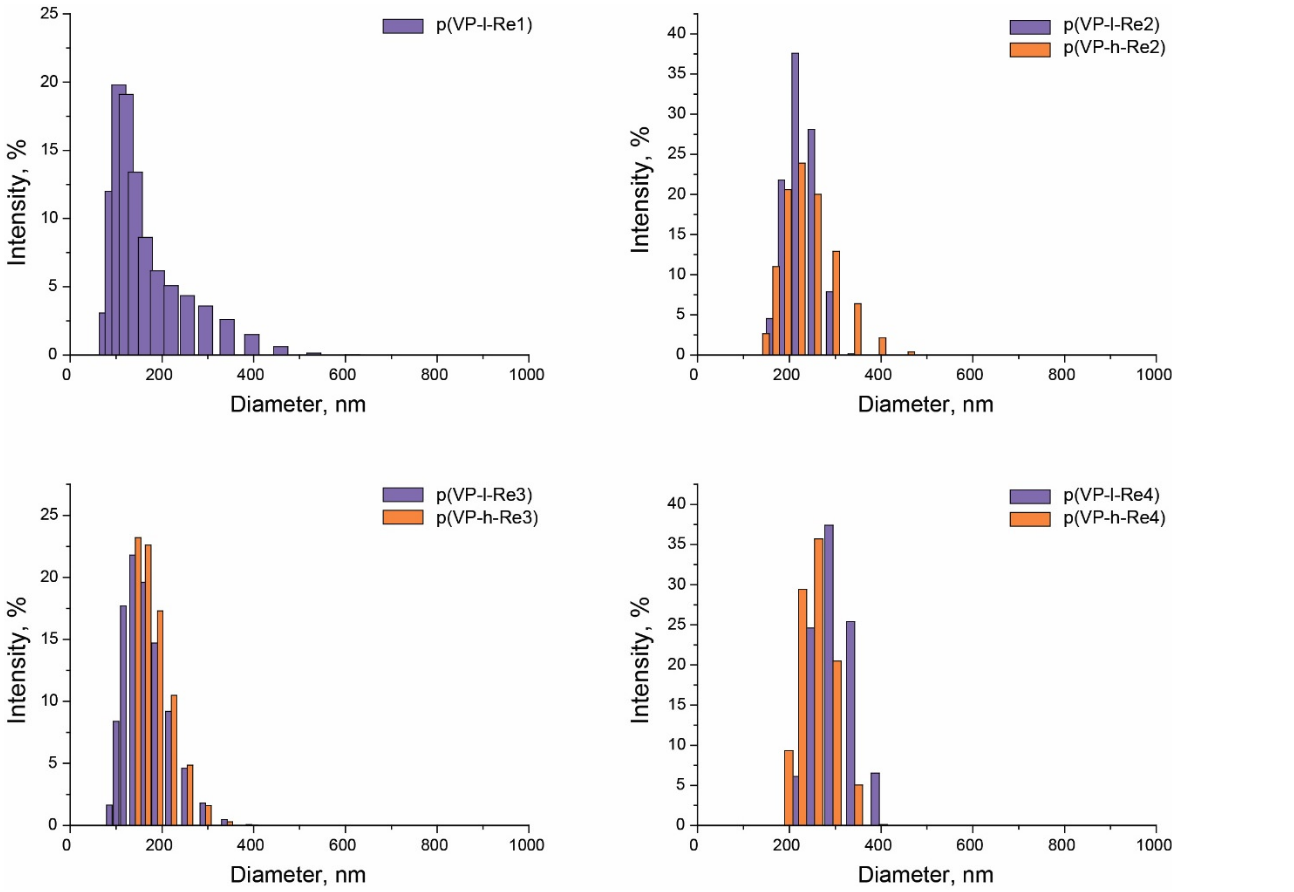

Figure S11. Particle sizes of the the **p(VP-l/h-Re)** polymers determined by dynamic light scattering in H_2O.

Part 3

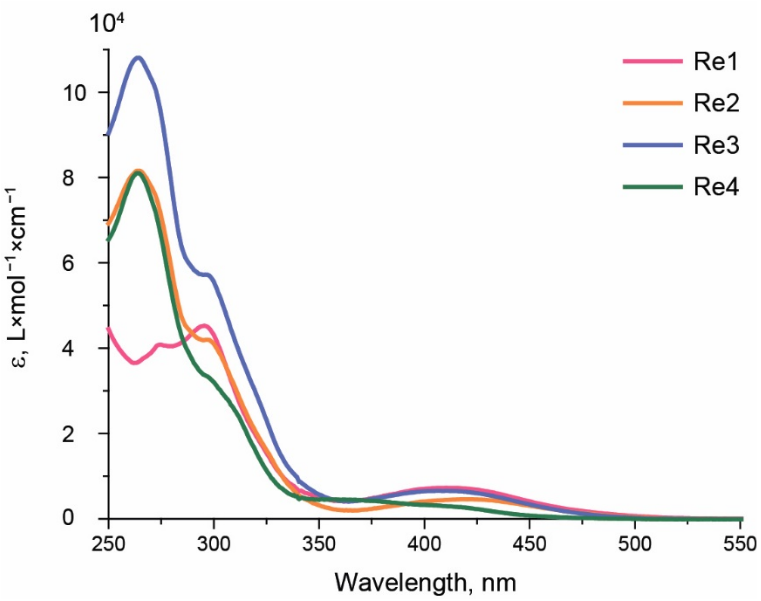

Figure S12. Electronic absorption spectra of complexes **Re1–Re4** in MeOH, 20°C.

Part 4

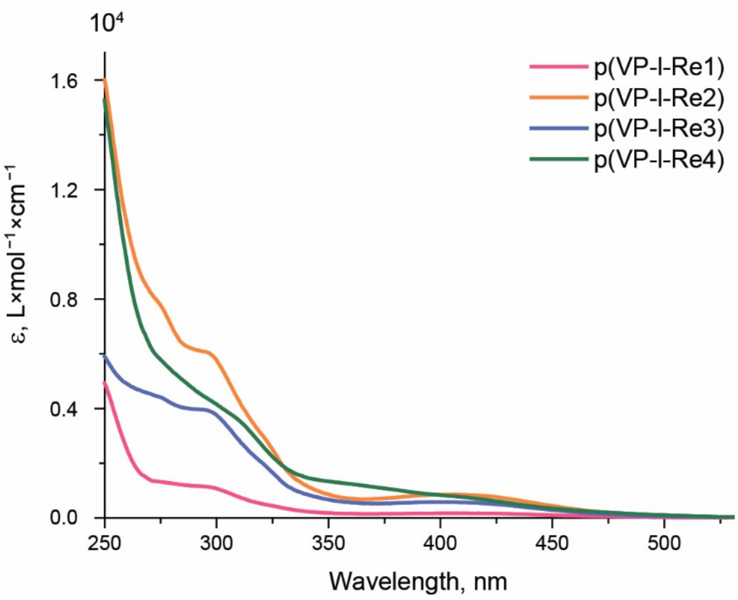

Figure S13. Electronic absorption spectra of **p(VP-l-Re)** in MeOH at 37°C.

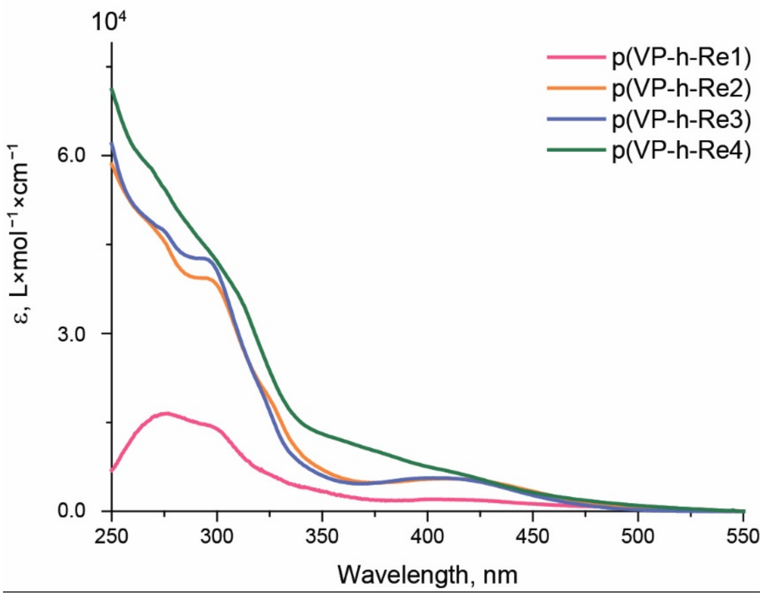

Figure S14. Electronic absorption spectra of **p(VP-h-Re)** in MeOH at 37°C.

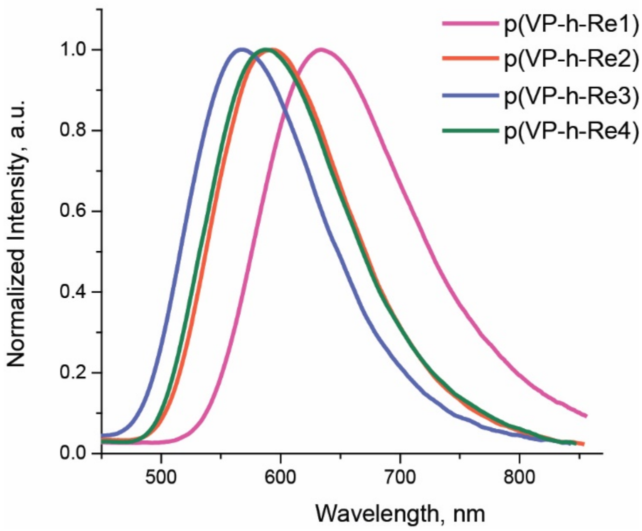

Figure S15. Emission spectra of **p(VP-h-Re)** in H_2O at 20°C.

PART 5

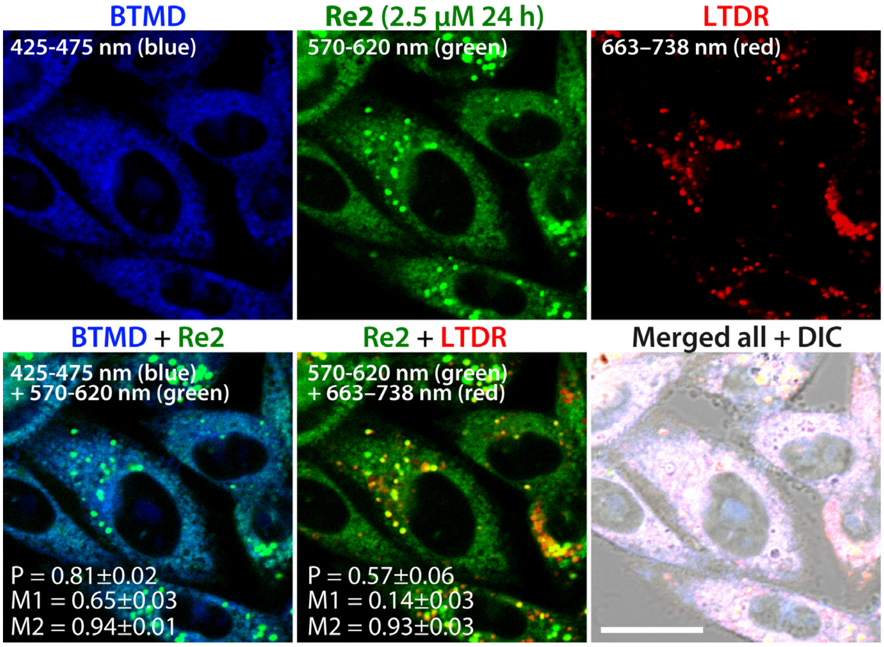

Figure S16**.** Subcellular distribution of BioTracker Blue Mitochondria Dye (BTMD, blue, 425-475 nm), **Re2** (green, 570-620 nm, 2.5 µM, 24 h), LysoTracker Deep Red (LTDR, red) in living CHO-K1 cells. Pearson's (P) and Manders' overlap coefficients (M1 – fraction of first channel that overlaps second channel, M2 – fraction of second channel that overlaps first channel) are presented as mean ± standard deviation calculated for 25 cells. Scale bar 20 µm.
